# Impact of initial dialysis modality on the survival of patients with ESRD: a propensity-score-matched study

**DOI:** 10.1186/s12882-023-03312-0

**Published:** 2023-10-26

**Authors:** Li Liu, Jie Pang, Juan Xu, Lin-na Liu, Man-yu Liao, Qing-xiu Huang, Yan-lin Li

**Affiliations:** Department of Nephrology, Zhongshan Hospital of Traditional Chinese Medicine Affiliated to Guangzhou University of Traditional Chinese Medicine, 3rd Kangxin Road, Zhongshan, 528400 China

**Keywords:** Hemodialysis, Peritoneal dialysis, Mortality, End-stage renal disease, Propensity score matching

## Abstract

**Background:**

Studies comparing the survival of hemodialysis (HD) and peritoneal dialysis (PD) patients are controversial. This study evaluated the impact of initial dialysis modality on the survival of patients with end-stage renal disease (ESRD) in a matched-pair cohort.

**Methods:**

A retrospective cohort study was performed on ESRD patients who initiated renal replacement treatment between January 1, 2010, and December 31, 2018. Propensity score matching was applied to balance the baseline conditions, and multivariate Cox regression analysis was applied to compare mortality between HD and PD patients and evaluate correlations between mortality and various baseline characteristics. Subgroup analysis was performed with respect to diabetes status.

**Results:**

There were 739 patients in our center in the Chinese National Renal Data System (CNRDS) between 2010 and 2018. Of these, 125 PD patients were matched with 125 HD patients. The 1-, 2-, and 3-year survival rates were 96.5%, 90.7%, and 82.5%, respectively, in the HD group and 99.5%, 97.8%, and 92.5%, respectively, in the PD group (log-rank P < 0.001). Among the propensity score-matched cohorts, no significant differences in Kaplan–Meier curves were observed between the two groups (log-rank P = 0.514). Age at dialysis initiation, CCI, congestive heart failure and cerebrovascular disease were risk factors in the multivariable-adjusted model. In subgroups defined by diabetes status, the Kaplan‒Meier survival curve showed that PD survival was significantly higher than that of HD (log-rank P = 0.022).

**Conclusions:**

HD and PD were not significantly different regarding the survival of patients with ESRD. PD was associated with better survival in diabetic ESRD patients.

## Introduction

An increasing incidence of end-stage renal disease (ESRD) has caused a substantial increase in the number of patients requiring renal replacement therapy (RRT) [1]. Hemodialysis (HD) and peritoneal dialysis (PD) are two common forms of dialysis therapy for ESRD [2].

Whether there exists a survival advantage for either HD or PD has been an area of intense interest and controversy over the past few years. Randomized controlled trials (RCTs) to assess the independent effect of HD and PD on survival have been impossible to conduct thus far [3]. Dialysis modality selection is generally based on consideration of all aspects by the patient and physician. Several observational studies have demonstrated that there is no difference in survival between dialysis modalities [4–7]. Some studies have shown that HD is associated with better survival [8], while some studies indicate that PD patients have better survival [9–11]. Recently, a survival advantage associated with PD has been reported in younger ESRD patients, and a survival advantage associated with HD has been reported in older patients [12]. However, in general, comparative mortality studies on this subject remain controversial. There is a need to further clarify whether the initial dialysis modality may impact survival.

Propensity-score-matched analysis reduces bias resulting from the nonrandom nature of the treatment assignment seen in observational studies [13]. Therefore, we conducted a study in our center to evaluate the impact of the initial dialysis modality on the survival of patients with ESRD by using a propensity-score-matched cohort.

## Materials and methods

### Study cohort

The study was approved by the institutional review board of Zhongshan Hospital of Traditional Chinese Medicine Affiliated to Guangzhou University of Traditional Chinese Medicine (approval No.2022ZSZY-LLK-453) and complied with the Declaration of Helsinki. The requirement for informed consent was waived because the study was retrospective. This retrospective cohort study was performed with all patients in the Chinese National Renal Data System (CNRDS) who initiated maintenance dialysis in our center between January 1, 2010, and December 31, 2018. The inclusion criterion was judged by a clinician. Patients were excluded for the following reasons: lack of baseline data, younger than 18 years old, follow-up less than 3 months, and other dialysis methods for more than 3 months before enrollment. Full-time staff were responsible for the system information registration and all of the maintenance dialysis patients’ follow-up in our center. Hence, the data of the cohort were relatively complete and reliable.

### Data collection

Data on baseline demographics, comorbid conditions, and laboratory test results were obtained by retrieved from our inpatient system and then compared with the data from the CNRDS. Demographic data included birth, sex, start of dialysis, and primary kidney disease. Comorbidities were identified at baseline by the International Classification of Diseases, 9th and 10th Revision (ICD-9 and ICD-10) codes, and the Charlson comorbidity index (CCI) was calculated based on Quan et al.’s method [14]. Laboratory indicators included blood urea nitrogen, serum creatinine, triglycerides, cholesterol, plasma albumin and hemoglobin.

### Outcomes and exposures

The outcome data were retrospectively retrieved from the CNRDS and inpatient systems. The main outcome was all-cause mortality. The censoring events included switching to another dialysis modality, undergoing a kidney transplant, transferring to another dialysis center or reaching the end of follow-up (December 31, 2021). The secondary outcomes were main adverse cardiovascular and cerebrovascular events (MACCE) and hospitalization. In this study, the MACCEs included cerebral hemorrhage, stoke, heart failure, myocardial infarction, unstable angina, peripheral vascular events and sudden death [15].

### Statistical analysis

All statistical analyses were performed using IBM SPSS Statistics software version 25.0 (IBM Corporation, Armonk, NY, USA) and GraphPad Prism 9 software (GraphPad Software, San Diego, CA, USA). Continuous variables that were normally distributed are presented as the mean ± standard deviation (SD), and t tests were used for comparison. Nonnormally distributed variables are presented as the median and rank, and the Mann‒Whitney U test was used for comparisons between groups. Categorical variables are presented as numbers (percentages) and were analyzed by the chi-square test. The Kaplan‒Meier survival curve was used to compare the overall survival between the initial dialysis modalities, and the significance of the difference was tested by the log-rank method.

Univariate and multivariate Cox proportional hazards regression models were used to compare the hazard ratios (HRs) with 95% confidence intervals (CIs) for death between the PD and HD patients, using the time from initial dialysis to censoring as the timescale. The results with a P value < 0.05 were considered statistically significant. Subgroup analysis was performed with respect to diabetes status.

Propensity score matching (PSM) was used to reduce selection bias to balance baseline status. The characteristics used in PSM were the same as the variables in the multivariate Cox regression model.

## Results

### Patients and data

**Fig. 1:**
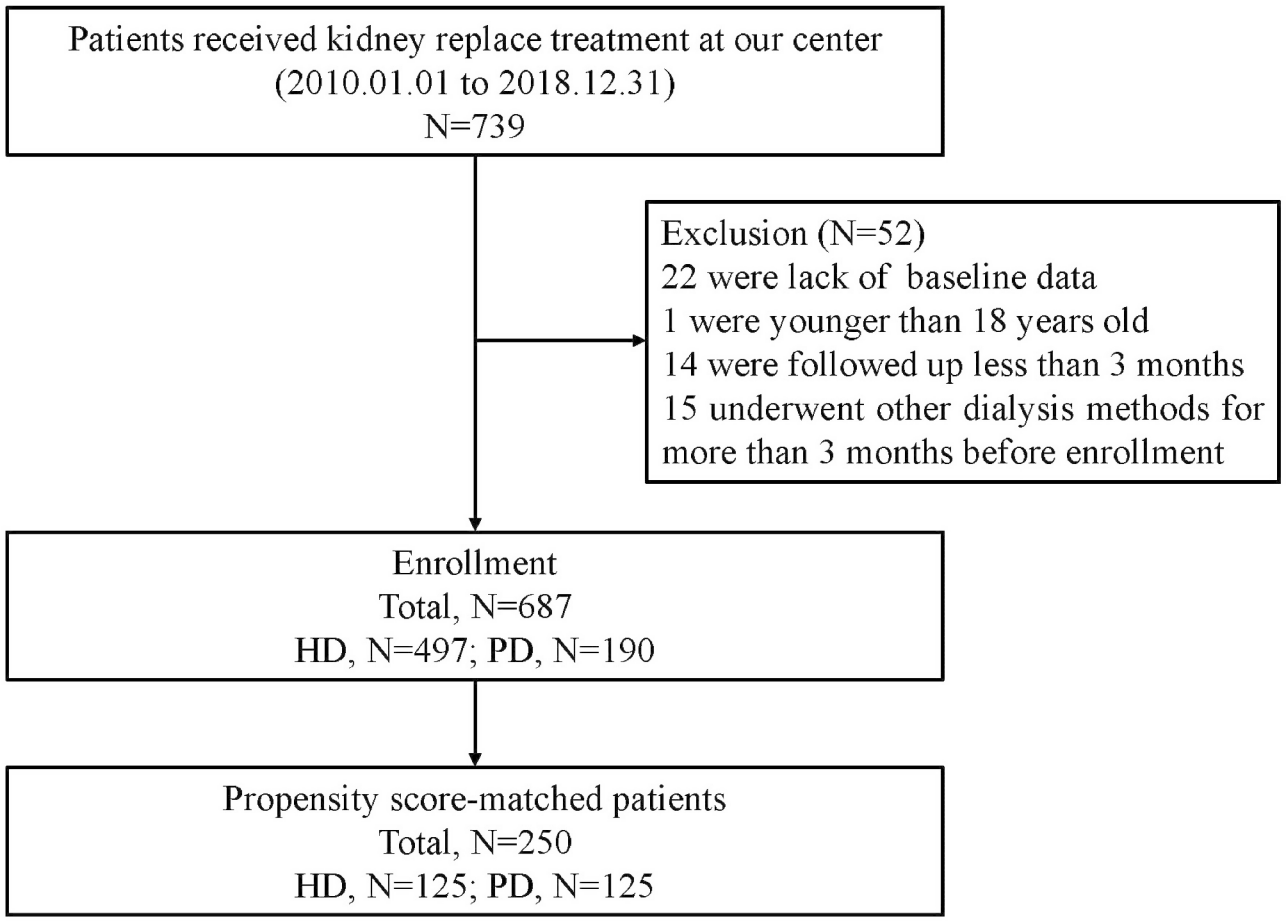
Study schematic *HD*, Haemodialysis; *PD*, Peritoneal dialysis

The study cohort profile is shown in Fig. 1. There were 739 patients in the CNRDS registered by our center during the study period. A total of 52 patients were excluded for the following reasons: lack of baseline data (n = 22), younger than 18 years old (n = 1), followed up less than 3 months (n = 14), and underwent other dialysis methods for more than 3 months before enrollment (n = 15). Among the 687 patients included, 497 had undergone HD, and 190 had undergone PD.

A propensity score was calculated in this cohort, and patients who were initially treated with HD were propensity score matched 1:1 with those who started with PD. A total of 125 matched pairs of patients were included in the final analyses. There were 60 patients with diabetes and 190 patients without diabetes among the cohort after matching.

### Patient characteristics at baseline

The baseline characteristics are shown in Table 1. The unmatched case-mix differences between HD and PD patients were significant. HD patients were older (56.2 ± 16.0 vs. 47.6 ± 13.5, *P* < 0.001) and presented a higher CCI value (5.4 ± 2.1 vs. 4.1 ± 1.7, *P* < 0.001) than PD patients. Compared with PD patients, HD patients also had higher rates of diabetes, congestive heart failure and cerebrovascular disease. In terms of kidney primary disease, the rate of obstructive nephropathy was higher in the PD group. Regarding laboratory tests, blood urea nitrogen and plasma albumin were higher in the HD group, while cholesterol showed the opposite trend.

**Table 1:** Baseline characteristics by modality at dialysis initiation before and after matching

Characteristics	Before Matching			After Matching		
	All HD (n = 497)	All PD (n = 190)	*P*	Matched HD (n = 125)	Matched PD (n = 125)	*P*
Demographic data						
Female (n)	188 (37.83%)	78 (41.05%)	0.44	49 (39.2%)	52 (41.6%)	0.70
Age at dialysis initiation (years)	56.2 ± 16.0	47.6 ± 13.5	**<0.01**	50.0 ± 15.5	49.6 ± 14.0	0.82
Duration of follow up (months)	54.6 (36.7, 81.1)	56.78 (39.8, 76.7)	0.83	54.4 (38.3, 90.8)	56.6 (39.9, 75.1)	0.77
Kidney primary disease						
Diabetic nephropathy (n)	114 (22.94%)	22 (11.58%)	0.05	14 (11.2%)	17 (13.6%)	0.57
Glomerulus nephritis (n)	188 (37.83%)	53 (27.89%)	0.94	42 (33.6%)	40 (32.0%)	0.79
Polycystic kidney (n)	14 (2.82%)	4 (2.11%)	0.99	3 (2.4%)	4 (3.2%)	1.00
Obstructive nephropathy (n)	29 (5.83%)	19 (10.00%)	**<0.01**	12 (9.6%)	10 (8.0%)	0.66
Other or unknown (n)	152 (30.58%)	92 (48.42%)	**<0.01**	54 (43.2%)	54 (43.2%)	1.00
Comorbidities						
Charlson Comorbidities Index (CCI)	5.4 ± 2.1	4.1 ± 1.7	**<0.01**	4.6 ± 2.0	4.4 ± 1.9	0.40
Diabetes (n)	171 (34.41%)	38 (20.00%)	**<0.01**	31 (24.8%)	29 (23.2%)	0.77
Cardiovascular disease (n)	144 (28.97%)	45 (23.68%)	0.16	33 (26.4%)	31 (24.8%)	0.77
Congestive heart failure (n)	104 (20.93%)	10 (5.26%)	**<0.01**	6 (4.8%)	9 (7.2%)	0.42
Cerebrovascular disease (n)	104 (20.93%)	12 (6.32%)	**<0.01**	17 (13.6%)	12 (9.6%)	0.32
Chronic pulmonary disease (n)	50 (10.06%)	36 (18.95%)	**<0.01**	22 (17.6%)	19 (15.2%)	0.61
Laboratory tests						
Serum Urea (mmol/L)	28.3 ± 8.3	22.7 ± 9.1	**<0.01**	24.9 ± 6.5	25.4 ± 9.2	0.62
Serum creatinine (umol/L)	977 (798, 1227)	972 (779, 1165)	0.56	947 (787, 1158)	973 (818, 1169)	0.55
Triglyceride (mmol/L)	1.5 (1.0, 2.1)	1.5 (1.1, 2.2)	0.67	1.6 (1.1, 2.6)	1.5 (1.1, 2.3)	0.44
Cholesterol (mmol/L)	4.4 ± 1.3	5.2 ± 1.3	**<0.01**	4.9 ± 1.3	5.0 ± 1.2	0.77
Plasma albumin (g/L)	37.3 ± 4.1	35.8 ± 5.9	**<0.01**	37.1 ± 4.4	36.7 ± 4.5	0.52
Hemoglobin (g/L)	97.7 ± 22.0	96.6 ± 23.7	0.28	96.4 ± 21.3	97.0 ± 24.6	0.85

*HD* Hemodialysis, *PD* Peritoneal dialysis

Bold values indicate significant statistical differences

After propensity score matching, 125 HD and 125 PD patients had similar characteristics, which suggested that these patients were likely eligible for either modality (Table 1).

Diabetes patients were included in the secondary analysis. As shown in Table 2, there were no significant differences in sex, age at dialysis initiation, or comorbidities between HD and PD patients. However, compared with PD patients, HD patients had a shorter duration of follow-up, higher rates of glomerulus nephritis, and lower values of serum creatinine and cholesterol.

**Table 2:** Baseline characteristics of diabetes patients in HD and PD groups after matching

Characteristics	HD (n = 31)		PD (n = 29)	*P*
Demographic data				
Female (n)	11 (35.50%)		10 (34.50%)	0.94
Age at dialysis initiation (years)	60.0 ± 11.7		54.6 ± 10.3	0.06
Duration of follow up (months)	40.0 (31.0, 49.1)		54.0 (46.5, 61.6)	**0.02**
Kidney primary disease				
Diabetic nephropathy (n)	12 (38.70%)		16 (55.20%)	0.20
Glomerulus nephritis (n)	8 (25.80%)		0 (0.00%)	**<0.01**
Polycystic kidney (n)	1 (3.20%)		0 (0.00%)	0.33
Obstructive nephropathy (n)	2 (6.50%)		4 (13.80%)	0.34
Other or unknown (n)	8 (25.80%)		9 (31.00%)	0.65
Comorbidities				
Charlson Comorbidities Index (CCI)	6.4 ± 1.8		6.1 ± 1.3	0.54
Cardiovascular disease (n)	13 (41.90%)		10 (34.50%)	0.55
Congestive heart failure (n)	2 (6.50%)		3 (10.30%)	0.59
Cerebrovascular disease (n)	9 (29.00%)		5 (17.20%)	0.28
Chronic pulmonary disease (n)	6 (19.40%)		9 (31.0%)	0.30
Laboratory tests				
Serum Urea (mmol/L)	22.9 ± 6.6		25.7 ± 8.9	0.17
Serum creatinine (umol/L)	814 (769, 859)		970 (839, 1101)	**0.02**
Triglyceride (mmol/L)	2.4 (1.9, 2.9)		2.3 (1.7, 2.9)	0.80
Cholesterol (mmol/L)	5.0 ± 1.1		5.6 ± 0.9	**0.04**
Plasma albumin (g/L)	35.5 ± 4.7		34.7 ± 4.9	0.50
Hemoglobin (g/L)	95.4 ± 18.3		97.5 ± 20.5	0.67

*HD* Hemodialysis, *PD* Peritoneal dialysis

Bold values indicate significant statistical differences

### Patient-level outcomes

#### Overall survival

**Fig. 2:**
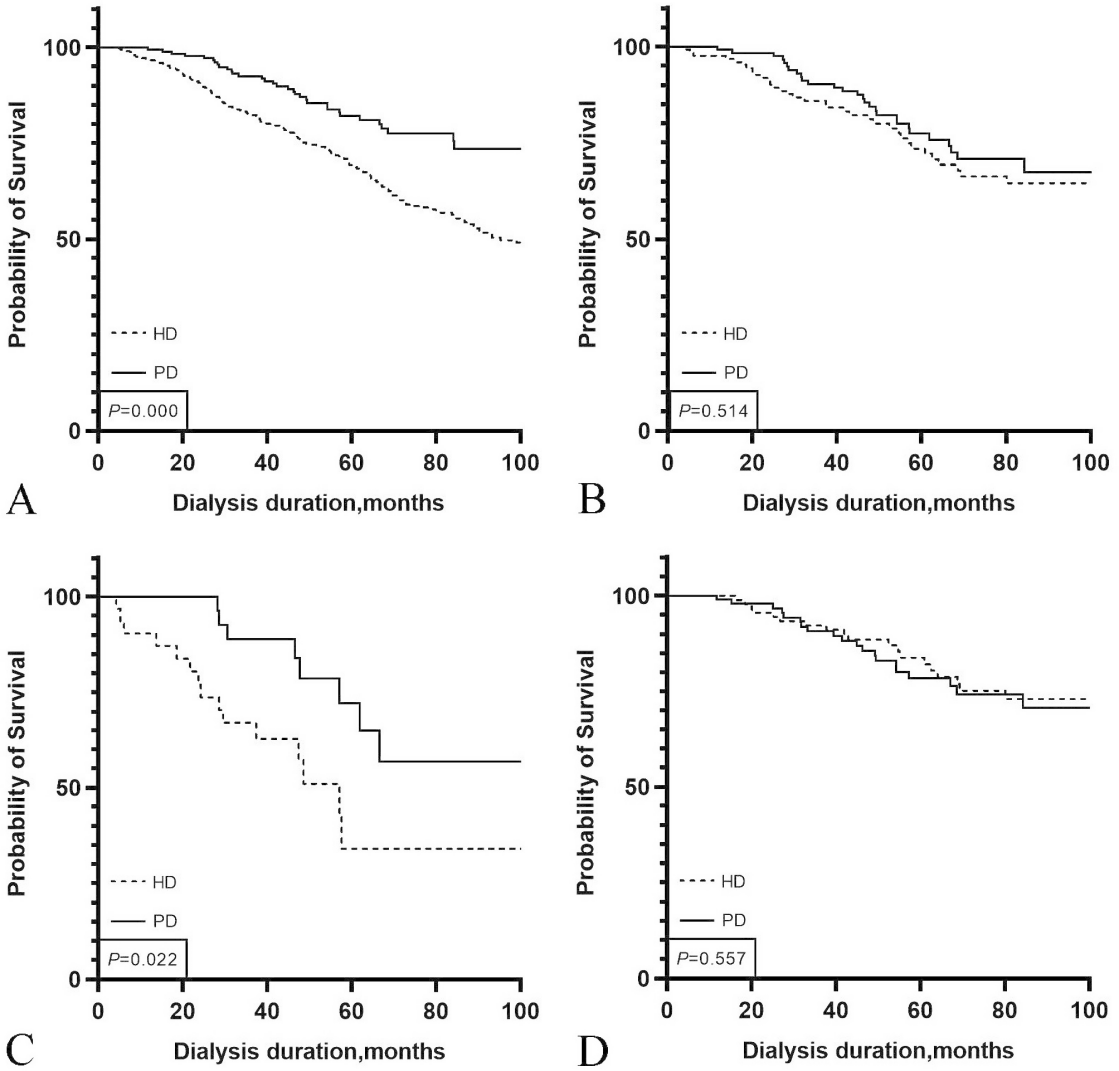
Comparison of survival rate between hemodialysis and peritoneal dialysis A: patients before matching; B: patients after PSM; C: diabetes group after PSM; D: no diabetes group after PSM. *HD*, Haemodialysis; *PD*, Peritoneal dialysis; *PSM*, Propensity score matching

The median follow-up period was 62.5 months for the HD patients and 75.7 months for the PD patients. A total of 217 (31.6%) death events occurred in the whole study cohort. The 1-, 2-, and 3-year survival rates were 96.5%, 90.7%, and 82.5%, respectively, in the HD group and 99.5%, 97.8%, and 92.5%, respectively, in the PD group (log-rank P < 0.001, Fig. 2A). Among the propensity score-matched cohorts, a total of 64 (25.6%) death events occurred. As shown in Table 3, the exposure-adjusted mortality was 5.3 per 100 patient-years in the HD group and 4.7 per 100 patient-years in the PD group (P = 0.538). No significant differences in Kaplan–Meier curves were observed between the two groups (log-rank P = 0.514, Fig. 2B).

**Table 3:** Patient-level outcomes of HD patients and matched PD patients

Events	HD (n = 125, 665 patient-years)		PD (n = 125, 612 patient-years)		P^*^
	No. of Events	Exposure-Adjusted Rate(per 100 patient-year)^#^	No. of Events	Exposure-Adjusted Rate(per 100 patient-year)^#^	
Death	35	5.3	29	4.7	0.538
MACCE	25	3.8	45	7.4	**< 0.001**
Hospitalization	268	40.3	205	33.5	**0.002**

*MACCE*, Main adverse cardiovascular and cerebrovascular events; *HD* Hemodialysis, *PD* Peritoneal dialysis

# The exposure-adjusted rate was calculated as 100 times the total number of events divided by the total number of patient-years of exposure

* P values were calculated for the exposure-adjusted incidence rate

#### Factors associated with survival

In the total study cohort, the univariate Cox regression model suggested that age at dialysis initiation, CCI, diabetes, cardiovascular disease, congestive heart failure and cerebrovascular disease were risk factors for all-cause mortality. Age at dialysis initiation, CCI, congestive heart failure and cerebrovascular disease were also risk factors in the multivariable-adjusted model. Age at dialysis initiation and cerebrovascular disease remained risk factors in multivariate Cox regression after using propensity scores to eliminate the differences in baseline characteristics. The HR of cerebrovascular disease increased from 1.94 (95% CI: 1.42–2.64) to 2.36 (95% CI: 1.19–4.69). Both univariate and multivariate Cox regression suggested that higher hemoglobin and female sex were protective factors. The HR of females decreased from 0.64 (95% CI: 0.48–0.86) to 0.41 (95% CI: 0.23–0.74) after propensity score matching (Table 4).

**Table 4:** Risk factors for mortality assessed by univariate and multivariate Cox regression model

Variables	Univariate (n = 687)			Multivariate ^a^ (n = 687)			PSM ^b^-Multivariate ^a^ (n = 250)	
	HR (95% CI)	*P*	HR (95% CI)		*P*	HR (95% CI)		*P*
Sex (female vs. male)	0.67 (0.51–0.89)	**0.01**	0.64 (0.48–0.86)		**<0.01**	0.41 (0.23–0.74)		**<0.01**
Age at dialysis initiation (≥ 65 years)	3.92 (2.99–5.15)	**<0.01**	2.35 (1.71–3.23)		**<0.01**	2.56 (1.26–5.18)		**0.01**
CCI (≥ 5)	4.11 (2.97–5.69)	**<0.01**	1.77 (1.17–2.68)		**0.01**	1.20 (0.60–2.39)		0.60
Diabetes	2.46 (1.87–3.23)	**<0.01**	1.32 (0.89–1.96)		0.16	1.43 (0.69–2.98)		0.34
Cardiovascular disease	1.40 (1.04–1.87)	**0.03**	1.23 (0.88–1.70)		0.22	0.88 (0.47–1.64)		0.68
Congestive heart failure	2.19 (1.61–2.98)	**<0.01**	1.77 (1.25–2.51)		**<0.01**	1.34 (0.54–3.33)		0.53
Cerebrovascular disease	3.16 (2.37–4.22)	**<0.01**	1.94 (1.42–2.64)		**<0.01**	2.36 (1.19–4.69)		**0.01**
Triglyceride (mmol/L)	0.88 (0.78–0.99)	**0.04**	0.98 (0.86–1.12)		0.81	1.00 (0.81–1.24)		0.99
Plasma albumin (g/L)	0.95 (0.93–0.98)	**<0.01**	0.98 (0.95–1.01)		0.21	0.98 (0.93–1.04)		0.55
Hemoglobin (g/L)	0.98 (0.97–0.99)	**<0.01**	0.98 (0.97–0.99)		**<0.01**	0.97 (0.96–0.98)		**<0.01**
Dialysis methods (HD vs. PD)	2.06 (1.43–2.97)	**<0.01**	1.09 (0.73–1.64)		0.68	1.04 (0.61–1.78)		0.89

*PSM*, Propensity scoring matching; *HR*, Hazard ratio; *95% CI*, 95% confidence interval; *CCI*, Charlson comorbidities index; *HD*, Hemodialysis; *PD*, Peritoneal dialysis

^a^ Variables adjusted in multivariate Cox regression model: Sex, Age at dialysis initiation, CCI, Diabetes, Cardiovascular disease, Congestive heart failure, Cerebrovascular disease, Triglyceride, Plasma albumin, Hemoglobin, Dialysis methods

^b^ The characteristics used in PSM were the same as the variables in the multivariate Cox regression model

Bold values indicate significant statistical differences

#### Subgroup analyses by diabetes status

According to the interaction effect analysis, all patients after PSM were divided into two groups based on diabetes status. In the diabetes group, the all-cause mortality rate ratio of HD to PD was 2.665 (95% CI: 1.118–6.352). In the nondiabetes group, the all-cause mortality rate ratio of HD to PD was 0.831 (95% CI: 0.447–1.545).

In the diabetes group, the 1-, 2-, and 3-year survival rates of the PD group were 100.0, 100.0, and 88.9%, respectively, while those of the HD group were 90.3, 77.0, and 66.8%, respectively. The Kaplan‒Meier survival curve showed that the survival rate of PD was significantly higher than that of HD (log-rank P = 0.022, Fig. 2C). In the nondiabetes group, there was no significant difference in the survival rate between HD and PD patients (Fig. 2D).

#### Main adverse cardiovascular and cerebrovascular events (MACCEs)

As shown in Table 3, the cumulative MACCE rates were 3.8 per 100 patient-years in the HD group and 7.4 per 100 patient-years in the matched PD group (P < 0.001). The number of MACCEs in the HD group was as follows: 69 episodes of heart failure, 10 episodes of stroke, 2 cases of unstable angina, 8 myocardial infarctions, and 4 other events. In the matched PD group, the number of MACCEs was as follows: 19 episodes of stroke, 131 episodes of heart failure, 1 case of unstable angina, 4 myocardial infarctions, and 3 other events.

The crude HR and adjusted HR of the occurrence of first MACCE in the HD patients compared with the PD patients were 0.856 (95% CI 0.587–1.249) and 0.834 (95% CI 0.567–1.226), respectively.

**Fig. 3:**
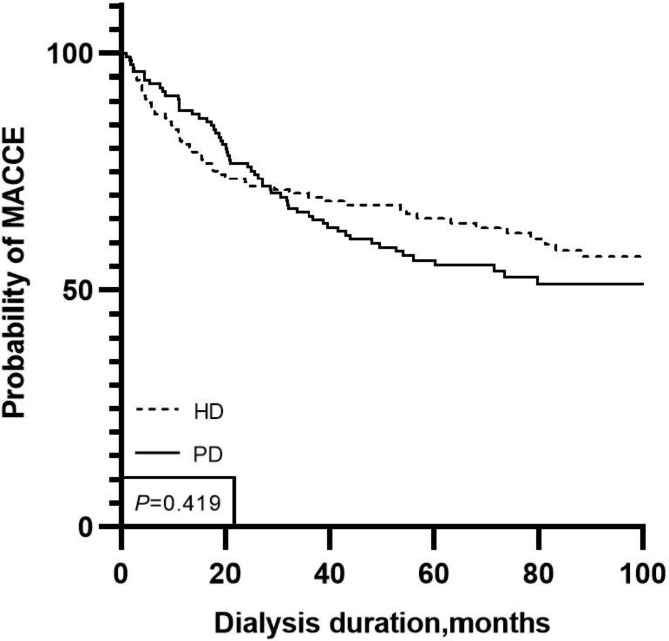
Kaplan-Meier MACCE-free estimate for the hemodialysis and peritoneal patients MACCE, Main adverse cardiovascular and cerebrovascular events; *HD*, Haemodialysis; *PD*, Peritoneal dialysis; *PSM*, Propensity score matching

The Kaplan–Meier survival curve also showed that there was no difference between HD and PD patients in the occurrence of a first MACCE (log-rank P = 0.419) (Fig. 3).

#### Hospitalization

The cumulative hospitalization rates were 40.3 per 100 patient-years in the HD group and 33.5 per 100 patient-years in the matched PD group, and this was a nominally significant result (P = 0.002) (Table 3).

## Discussion

In this retrospective observational cohort study, we compared the impact of the initial dialysis modality on the survival of patients with ESRD beginning dialysis in our center between January 1, 2010, and December 31, 2018. Our study showed that mortality was significantly higher in patients initiating dialysis with HD than in those initiating dialysis with PD in the whole cohort. However, in the propensity score-matched cohort, there was no difference between HD and PD patients regarding survival and the occurrence of first MACCE. However, HD was demonstrated to be associated with decreased MACCE rates, while PD was demonstrated to be associated with decreased cumulative hospitalization rates. We also found that PD was more favorable with respect to survival than HD in patients with diabetes.

Randomized controlled trials assessing the independent effect of HD and PD on survival have been impossible to conduct [3]. Several studies reported that there is no difference in survival between the modalities [4–7], which is consistent with our findings. Several studies also reported favorable outcomes of PD in younger patients or during the first 1–2 years of dialysis treatment compared to the outcomes of HD [12, 16, 17]. We did not find any interaction between age and dialysis modality related to outcomes, and the concern about increased mortality in PD patients if treatment is continued beyond 1–2 years was also not supported by our study.

In another subgroup analysis of our matched cohort, significantly better survival was shown in patients with diabetes whose treatment was initiated with PD versus those initiating with HD. This is contradictory to the results of several studies, which have claimed that patients with diabetes mellitus did worse on PD than on HD [18, 19], while favorable outcomes of PD were reported in patients without diabetes [10, 20]. It was generally recognized in previous studies that PD therapy may affect blood glucose control in ESRD patients because the dialysate used for PD contains glucose [21–23], and diabetic patients are prone to developing disorders of lipid metabolism [24], which might accelerate the process of arteriosclerosis and increase the incidence of cardiovascular events [25, 26]. There were also some studies that found no interactions between diabetes mellitus and initial modality concerning mortality [9, 10, 27].

There are several reasons why our results may diverge from those of previous studies comparing outcomes in PD and HD in patients with diabetes. First, we speculate therapy skills, including elective dialysate and automated peritoneal dialysis (APD) prescription while avoiding glucose load, might have been responsible for our favorable results. Regretfully, we did not include PD prescriptions in this retrospective study. Further research is needed to verify this speculation. Second, the team responsible for training, management and follow-up of PD patients was of high quality in our single center, which might account for the different results of this single center study compared to other studies. Moreover, when PD fails, it is common to switch to HD; therefore, the mortality rate of these patients was lower than that of HD patients [10]. There were also reports consistent with our discovery that PD showed better outcomes in diabetes patients [28, 29].

With regard to the factors associated with survival, we found that age, cerebrovascular disease, and lower hemoglobin were risk factors, while female sex were protective factors, which are not totally consistent with those reported by other studies. Some studies [6, 30–32] discovered that age and diabetes were risk factors associated with death. Another study in eastern China [10] followed up 22,379 patients for a median of 29 months and found that age, diabetic nephropathy, and cardiovascular disease were risk factors. Overall, most studies found that age is a risk factor affecting survival in dialysis patients, which is consistent with our findings. Additionally, cerebrovascular disease and lower hemoglobin were also risk factors in this study. Anemia is a common complication among dialysis patients. The literature [33] states that adverse cardiovascular events and all-cause mortality among patients decrease by between 6% and 5%, respectively, for every 10 g/L increase in hemoglobin levels among dialysis patients. In addition, in patients with anemia, the use of high-dose erythropoietin may increase the risk of cerebrovascular disease by increasing hypertension, vascular sclerosis, and blood viscosity. The impact of gender on survival in dialysis patients is still under debate. Although some studies showed no difference in survival [6, 10], there were studies that revealed a decreased risk of technique failure for females [34, 35]. The survival advantage in female patients may be due to better compliance with standardized management, or more independence from others in maintaining their selfcare than male patients.

There are several limitations of this study worth mentioning. The main limitation is that it was not a randomized study but rather a retrospective observational cohort study, and propensity score matching can account only for observed confounders. Despite propensity score matching and adjustment for several confounding factors, residual confounding cannot be excluded. Therefore, this study may not be completely free of bias due to confounding. Moreover, we failed to use 1:2 propensity score matching, which will be more representative of the data. Second, we stopped follow-up when the dialysis modality change event occurred, and failed to collect complete data to conduct a sensitivity analysis. The effect of switching the type of RRT and the possibility of changing dialysis mode to reduce the short-term risk of death was not considered. Third, only baseline laboratory test results were recorded, which might be altered during dialysis treatment. Therefore, the laboratory tests shown in this study could not reflect the situation of patients in the treatment process. Fourth, the study population was small (250 patients), and the survival rates of only the first 3 years were compared. Last, only 60 diabetes patients were included in the secondary analysis, and the two groups were not well matched, as the HD patients had a shorter duration of follow-up, higher rates of glomerulus nephritis, and lower values of serum creatinine and cholesterol.

## Conclusion

In conclusion, our study showed that the two modes of dialysis were not significantly different regarding the survival of patients with ESRD. PD was associated with better survival in diabetic ESRD patients. More research is needed to verify whether PD may be a therapy of priority in diabetic ESRD patients.

## Data Availability

The datasets used and/or analyzed during the current study are available from the corresponding author on reasonable request and with permission of Zhongshan Hospital of Traditional Chinese Medicine Affiliated to Guangzhou University of Traditional Chinese Medicine.

## Abbreviations

ESRD: End-stage renal disease
HD: Hemodialysis
PD: Peritoneal dialysis
RCT: Randomized controlled trial
CCI: Charlson comorbidities index
CNRDS: Chinese National Renal Data System
SD: Standard deviation
PSM: Propensity score matching
HR: Hazard ratio
CI: Confidence interval
APD: Automated peritoneal dialysis
